# COVID-19 Vaccination reduced pneumonia severity^☆^

**DOI:** 10.1016/j.ejro.2022.100456

**Published:** 2022-11-11

**Authors:** Noriaki Wada, Yi Li, Takuya Hino, Staci Gagne, Vladimir I. Valtchinov, Elizabeth Gay, Mizuki Nishino, Bruno Madore, Charles R.G. Guttmann, Sheila Bond, Kousei Ishigami, Gary M. Hunninghake, Bruce D. Levy, Kenneth M. Kaye, David C. Christiani, Hiroto Hatabu

**Affiliations:** a Center for Pulmonary Functional Imaging, Department of Radiology, Brigham and Women’s Hospital and Harvard Medical School, 75 Francis Street, Boston, MA 02115, USA; b Department of Biostatistics, University of Michigan, 1415 Washington Heights, Ann Arbor, MI 48109, USA; c Department of Clinical Radiology, Graduate School of Medical Sciences, Kyushu University, 3–1-1 Maidashi, Higashi-ku, Fukuoka, Fukuoka 8128582, Japan; d Pulmonary and Critical Care Medicine, Brigham and Women’s Hospital and Harvard Medical School, 75 Francis Street, Boston, MA 02115, USA; e Center for Neurological Imaging, Department of Radiology, Brigham and Women’s Hospital and Harvard Medical School, 75 Francis Street, Boston, MA 02115, USA; f Division of Infectious Diseases, Brigham and Women’s Hospital and Harvard Medical School, 75 Francis Street, Boston, MA 02115, USA; g Pulmonary and Critical Care Division, Department of Medicine, Massachusetts General Hospital and Harvard Medical School, 55 Fruit Street, Boston, MA. 02114, United States; h Department of Environmental Health, Harvard TH Chan School of Public Health, 655 Huntington Avenue, Boston, MA 02115, USA

**Keywords:** COVID-19, coronavirus disease 2019, SARS-CoV-2, severe acute respiratory syndrome coronavirus 2, RT-PCR, reverse transcription-polymerase chain reaction, mRNA, messenger ribonucleic acid, GGO, ground-glass opacity, OP, organizing pneumonia, DAD, diffuse alveolar damage, COVID-19, Pneumonia, CT, Vaccination, Lung

## Abstract

**Purpose:**

To investigate the effect of vaccinations and boosters on the severity of COVID-19 pneumonia on CT scans during the period of Delta and Omicron variants.

**Methods:**

Retrospectively studied were 303 patients diagnosed with COVID-19 between July 2021 and February 2022, who had obtained at least one CT scan within 6 weeks around the COVID-19 diagnosis (−2 to +4 weeks). The severity of pneumonia was evaluated with a 6-point scale Pneumonia Score. The association between demographic and clinical data and vaccination status (booster/additional vaccination, complete vaccination and un-vaccination) and the difference between Pneumonia Scores by vaccination status were investigated.

**Results:**

Of 303 patients (59.4 ± 16.3 years; 178 females), 62 (20 %) were in the booster/additional vaccination group, 117 (39 %) in the complete vaccination group, and 124 (41 %) in the unvaccinated group. Interobserver agreement of the Pneumonia Score was high (weighted kappa score = 0.875). Patients in the booster/additionally vaccinated group tended to be older (P = 0.0085) and have more underlying comorbidities (P < 0.0001), and the Pneumonia Scores were lower in the booster/additionally vaccinated [median 2 (IQR 0–4)] and completely vaccinated groups [median 3 (IQR 1–4)] than those in the unvaccinated group [median 4 (IQR 2–4)], respectively (P < 0.0001 and P < 0.0001, respectively). A multivariable linear analysis adjusted for confounding factors confirmed the difference.

**Conclusion:**

Vaccinated patients, with or without booster/additional vaccination, had milder COVID-19 pneumonia on CT scans than unvaccinated patients during the period of Delta and Omicron variants. This study supports the efficacy of the vaccine against COVID-19 from a radiological perspective.

## 1 Introduction

Coronavirus disease 2019 (COVID-19) vaccines are highly effective in reducing infection, severe disease, hospitalization, and death associated with COVID-19 [1], [2], [3], [4], [5]. However, the phenomenon of breakthrough infections has been frequently reported in persons who have completed a primary vaccination series [6], likely because of the waning immunity caused by loss of vaccine-induced immunological protection or lower vaccine effectiveness against new variants [7]. Newly implemented booster and additional vaccinations among adults were highly effective at preventing severe acute respiratory syndrome coronavirus 2 (SARS-CoV-2) infection or death during the Delta and Omicron variant predominant period [4], [8].

The acute to long-term findings on chest computed tomography (CT) and their associated clinical factors in COVID-19 patients have been previously reported [9], [10], [11], [12], [13]. Some recent studies showed that patients vaccinated against COVID-19 had milder CT pneumonia severity than unvaccinated patients [14], [15], [16]. However, there are limited reports about CT findings in patients who received a booster or additional vaccine dose [14], and of these reports, the findings have been inconclusive due to variant prevalence at the time of the study and the regional differences in vaccination methods.

To fill this critical gap, the present study investigates the association between the vaccination status and the maximum severity of pneumonia on CT scan obtained within a 6-week window around diagnosis (−2 to +4 weeks) during the Delta and Omicron variant predominant period. Our hypothesis is that full vaccination, with or without a booster/additional dose, is associated with milder pneumonia on CT scans obtained around the time of diagnosis.

## 2 Materials and methods

### 2.1 Patient selection, inclusion, and exclusion

This study was approved by the local institutional review board (IRB#2021P000981) and was performed in accordance with the principles of the Declaration of Helsinki. We retrospectively studied inpatients and outpatients diagnosed with COVID-19 by the positive reverse transcription-polymerase chain reaction (RT-PCR) test for SARS-CoV-2 between July 24, 2021, and February 17, 2022, corresponding to a circulation period predominantly composed of Delta and subsequent Omicron variants in the United States [5], [8]. The inclusion criteria consisted of at least one CT scan performed within a 6-week window around the time of the COVID-19 diagnosis (−2 to +4 weeks). The exclusion criteria were: (1) patients with unknown vaccination status or incomplete vaccination records, including series of product name and vaccination date; or (2) patients with only one dose of messenger ribonucleic acid (mRNA) vaccine [BNT162b2 (Pfizer-BioNTech) or mRNA-1273 (Moderna) vaccines].

### 2.2 Demographic and clinical data

Demographic data, including age, sex, body mass index (BMI), smoking status, and vaccination records, including product names and vaccination dates, were obtained from electronic medical records. Clinical data were collected, including the number of hospital stays, hospitalization, intensive care unit (ICU) admission, all-cause death, C-reactive protein (CRP), D-dimer, and underlying comorbidities such as lung disease, hypertension (HT), coronary artery disease (CAD), diabetes mellitus (DM), chronic kidney disease (CKD), and malignancy. Lung disease included asthma, chronic obstructive pulmonary disease, interstitial lung disease, lung cancer, and lung surgery.

### 2.3 Definition of vaccination status

Patients were categorized as unvaccinated, completely vaccinated, or booster or additionally vaccinated groups. Completely vaccinated referred to those who had received two doses of BNT162b2 or mRNA-1273 vaccine or one dose of adenovirus vector-based vaccine [Ad26. COV2. S (Johnson & Johnson-Janssen) vaccine] and at least 14 days had passed since the last vaccination. Booster or additionally vaccinated patients were those who had received three doses of BNT162b2 or mRNA-1273 vaccine, two doses of Ad26. COV2. S vaccine, or each one dose of Ad26. COV2. S vaccine and BNT162b2 or mRNA-1273 vaccine and at least 7 days had passed since the last vaccination. Each cutoff of 14 days and 7 days was set with reference to the previous studies [5] to ensure sufficient time for a completely- or booster-specific immune response. Booster vaccination referred to vaccination to enhance or restore protection by the primary vaccination, which might have waned over time, and additional vaccination refers to vaccination for moderately to severely immunocompromised persons who may not have mounted a protective immune response after primary vaccination [17]. Because it was difficult to distinguish between a booster dose and an additional dose in the electronic medical records, both were classified into the same group as the booster/additional dose group.

### 2.4 CT scan of Chest

Chest CT scan was performed using standard chest CT protocols at our institute with or without administration of the intravenous contrast. Images were reconstructed with a slice thickness from 1 to 3 mm, and all the section CT images were reviewed in a lung window: window width of 1000 to 2000 Hounsfield units; window level of − 700 to − 500 Hounsfield units.

### 2.5 Image evaluation and classification

CT scans obtained within the -2 to 4 weeks window around diagnosis were classified into a six-point-scale severity score (Pneumonia Score) based on the pattern of pneumonia and extent of anatomical involvement on chest CT, a modified method of the pattern categorization proposed by Jin et al. [18]; No Pneumonia (Estimated Extent, 0 %), 1: ground-glass opacity (GGO) (<10 %), 2: Transition from GGO to organizing pneumonia (OP) patten (10–20 %), 3: OP patten (20–40 %), 4: Extensive OP pattern (40–70 %), and 5: diffuse alveolar damage (DAD) patten (>70 %) (Table 1). When more than one CT scans were available in the six-week window, the Pneumonia Score was determined for all available scans and the highest score was selected. Two experienced thoracic radiologists (NW, HH) independently and carefully read the CT images and provided the score, which became the final score when the two readers agreed. When these two readers differed, another experienced and blinded thoracic radiologist (MN) determined the final score. All three radiologists were blinded to the demographical/clinical data of the patients. In addition, the interobserver agreement of Pneumonia Score was calculated between the first two readers in 100 cases.

**Table 1:** Pneumonia Score: CT severity score based on pattern and extent of COVID-19.

Pneumonia score	Pattern	Estimated extent^a^
**0**	No pneumonia	0 %
**1**	GGO	< 10 %
**2**	Transition from GGO to OP patten	10–20 %
**3**	OP patten^b^	20–40 %
**4**	Extensive OP pattern	40–70 %
**5**	DAD patten^c^	> 70 %

GGO, ground-glass opacity; OP, organizing pneumonia; DAD, diffuse alveolar damage

^a^ Extent was estimated based on the percentage of overall lung involvement bilaterally.

^b^ OP patten is characterized by multifocal bilateral parenchymal consolidation.

^c^ DAD pattern is characterized by diffuse or multifocal GGOs/consolidation predominantly in dependent lung region, may be accompanied by lung volume loss and traction bronchiectasis.

### 2.6 Statistical analyses

Numerical variables were presented as mean ± standard deviation for normally distributed data and median [interquartile range (IQR)] for non-normal data. Categorical variables were presented using percentage, and their associations with the vaccination status were assessed by using Fisher’s exact test. Age and BMI among the three groups were examined with one-way ANOVA, while CRP, D-dimer, and hospital stays were examined with the Kruskal–Wallis test. Multiple comparisons were adjusted by using the Bonferroni method. The Wilcoxon rank-sum test was used to determine differences between Pneumonia Scores by vaccination status. The Pneumonia Score was assessed with a multivariate linear regression analysis to confirm the presence or absence of confounding factors. All statistical analyses were performed using JMP Pro15.1.0 (SAS, Cary, NC, USA). A two-sided P value less than 0.05 was considered significant.

## 3 Results

### 3.1 Inclusion criteria of the cohort

**Fig. 1:**
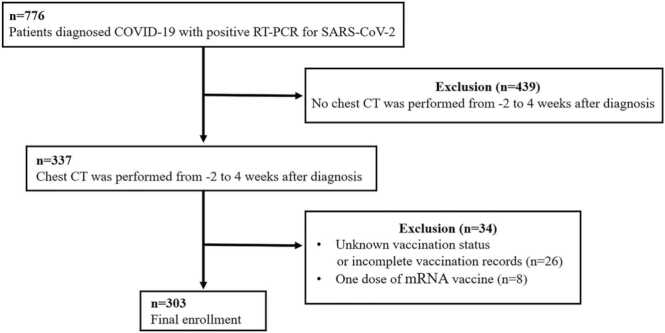
Flowchart of study selection based on inclusion and exclusion criteria.

Initially, we identified 776 cases with a diagnosis of COVID-19 based on a RT-PCR for SARS-CoV-2 with accessible chart information utilizing our institutional research patient data repository (RPDR). The flow chart for inclusion/exclusion criteria is shown in Fig. 1. Four hundred thirty-nine cases were excluded because no chest CT scan was performed within the six-week window around diagnosis. Among those with chest scans, thirty-four cases were excluded because of unknown vaccination status or incomplete vaccination records, such as no product names and vaccination date (n = 26), or with only one dose of mRNA vaccine (BNT162b2, n = 6, and mRNA-1273, n = 2). Ultimately, a total of 303 COVID-19 patients (59.4 ± 16.3 years; 178 females) were included and deemed analyzable in this study.

### 3.2 Demographic and clinical data

As patients without CT scans were excluded from the study, and to ascertain whether there was selection bias, we compared the vaccination status and the demographic and comorbidity conditions between those with and without chest scans (*see* Supplementary results).

**Fig. 2:**
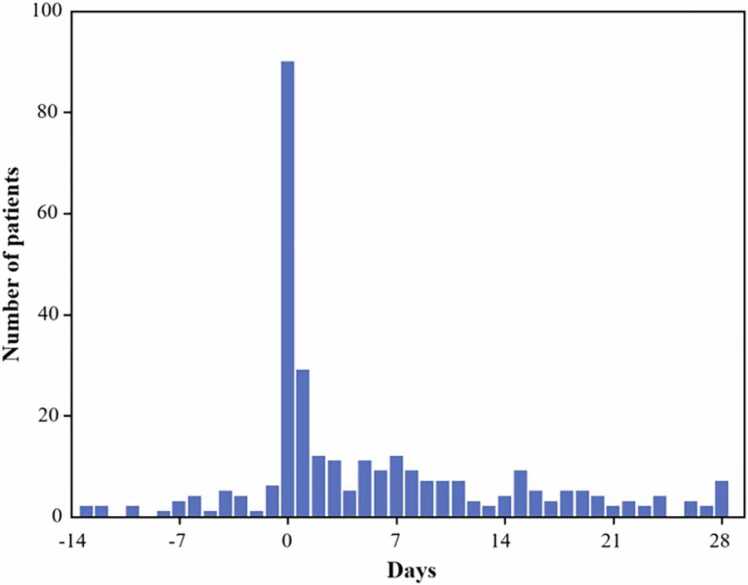
Histogram of the interval of time between diagnosis and CT with Pneumonia Score.

After exclusion, summarized vaccination, clinical and outcome data for the 303 analyzable patients are provided in Table S2. CT scans were performed within a 6-week window around the COVID-19 diagnosis (−2 to +4 weeks) [once, n = 247 (81 %); twice, n = 36 (12 %); three times, n = 17 (6 %); four times, n = 3 (1 %)]. The interval of time between diagnosis and CT with Pneumonia Score is summarized in Fig. 2, and the median interval was 2 (IQR 0–10) days.

Demographic and clinical data stratified by vaccination status are provided in Table 2. Of 303 patients, 62 (20 %) patients were in the booster or additional vaccination group, 117 (39 %) patients in the complete vaccination group, and 124 (41 %) patients in the unvaccinated group. The mean age was higher in the booster or additionally (63.6 ± 14.6 years) and completely (60.5 ± 15.5 years) vaccinated groups than in the unvaccinated group (56.2 ± 17.2 years) (P = 0.0085). The percentages of patients with smoking history were higher in the completely vaccinated group (58 %, 68/117) than in the unvaccinated group (37 %, 46/124) (P = 0.0043). The percentages of patients with at least one comorbidity were higher in the booster or additionally (98 %, 61/62) and completely (91 %, 107/117) vaccinated groups than in the unvaccinated group (70 %, 87/124) (P < 0.0001). The percentages of patients with lung disease were higher in the booster or additionally (52 %, 32/62) and completely (45 %, 53/117) vaccinated groups than in the unvaccinated group (23 %, 29/124) (P < 0.0001). The percentages of patients with HT and CAD were higher in the booster or additionally vaccinated group (66 %, 41/62 % and 21 %, 13/62, respectively) than in the unvaccinated group (47 %, 58/124 % and 6 %, 8/124, respectively) (P = 0.019 and P = 0.013, respectively). The percentages of patients with CKD were higher in the completely vaccinated group (16 %, 19/117) than in the unvaccinated group (6 %, 7/124) (P = 0.014). The percentages of patients with malignancy were higher in the booster or additionally vaccinated groups (56 %, 35/62) than in the completely vaccinated (31 %, 36/117) and unvaccinated (19 %, 24/124) group (P < 0.0001). The booster or additionally vaccinated groups [median 4 (IQR 0–9.3)] had shorter hospital stays than the unvaccinated group [median 6 (IQR 3–14)] (P = 0.035). The percentages of patients who required hospitalization and ICU admission were lower in the booster or additionally vaccinated group (65 %, 40/62 % and 8 %, 5/62, respectively) than in the unvaccinated group (84 %, 104/124 % and 24 %, 30/124, respectively) (P = 0.014 and P = 0.012, respectively). There was no difference in sex, BMI, CRP, D-dimer, DM, and all-cause death, among the three groups.

**Table 2:** Demographic data and clinical information stratified by vaccination status (n = 303).

	Unvaccinated (n = 124)	Completely vaccinated (n = 117)	Booster or additionally vaccinated (n = 62)	P value
Age (years)	56.2 ± 17.2	60.5 ± 15.5^a^	63.6 ± 14.6^b^	0.0085
Sex (Male:Female)	57:67	43:74	25:37	0.36
BMI (kg/m^2^)	31.5 ± 8.4	31.2 ± 10.3	28.7 ± 9.4	0.13
Smoking history Never Former/Current Unknown	72/124 (58 %) 46/124 (37 %) 6/124 (5 %)	46/117 (39 %)^a^ 68/117 (58 %)^a^ 3/117 (3 %)	26/62 (42 %) 34/62 (55 %) 2/62 (3 %)	0.0043^c^
Laboratory findings CRP (mg/L) (n = 210)	97 (43–197) (n = 98)	96 (48–160) (n = 75)	98 (15–175) (n = 37)	0.68
D-dimer (ng/mL) (n = 226)	1471 (846–3523) (n = 108)	1479 (806–3552) (n = 81)	1341 (525–3869) (n = 37)	0.91
Comorbidities Any comorbidity	87/124 (70 %)	107/117 (91 %)^a^	61/62 (98 %)^b^	< 0.0001
Lung disease	29/124 (23 %)	53/117 (45 %)^a^	32/62 (52 %)^b^	< 0.0001
HT	58/124 (47 %)	71/117 (61 %)	41/62 (66 %)^b^	0.019
CAD	8/124 (6 %)	16/117(14 %)	13/62 (21 %)^b^	0.013
DM	31/124 (25 %)	41/117 (35 %)	17/62 (27 %)	0.21
CKD	7/124 (6 %)	19/117 (16 %)^a^	10/62 (16 %)	0.014
Malignancy	24/124 (19 %)	36/117 (31 %)	35/62 (56 %)^c^^d^	< 0.0001
Clinical outcome				
Hospitalization	104/124 (84 %)	91/117 (78 %)	40/62 (65 %)^b^	0.014
Hospital stay (days)	6 (3–14)	5 (1–11.5)	4 (0–9.3)^b^	0.035
ICU admission	30/124 (24 %)	16/117 (14 %)	5/62 (8 %)^b^	0.012
All-cause death	19/124 (15 %)	22/117 (19 %)	13/62 (21 %)	0.59

BMI, body mass index; CRP, C-reactive protein; HT, hypertension; CAD, coronary artery disease; DM, diabetes mellitus; CKD, chronic kidney disease; ICU, intensive care unit

a The difference between unvaccinated and completely vaccinated patients was statistically significant (Bonferroni-adjusted P < 0.05).

b The difference between unvaccinated and booster or additionally vaccinated patients was statistically significant (Bonferroni-adjusted P < 0.05).

c Fisher’s exact test was performed using two variables: never and former/current smoking history.

d The difference between completely vaccinated and booster or additionally vaccinated patients was statistically significant (Bonferroni-adjusted P < 0.05).

### 3.3 Vaccination data

The median months from last vaccination of mRNA vaccine (BNT162b2 or mRNA-1273) and Ad26. COV2. S vaccine to diagnosis of COVID-19 were 7.5 (IQR 5.8–9.0) and 8.9 (IQR 7.3–9.9), respectively, in the completely vaccinated group and 2.6 (IQR 1.7–4.0) and 1.1 (IQR 0.3–2.0), respectively, in the booster or additionally vaccinated group. Details of vaccine type summarized in Tables S3 and S4.

### 3.4 Pneumonia score

**Fig. 3:**
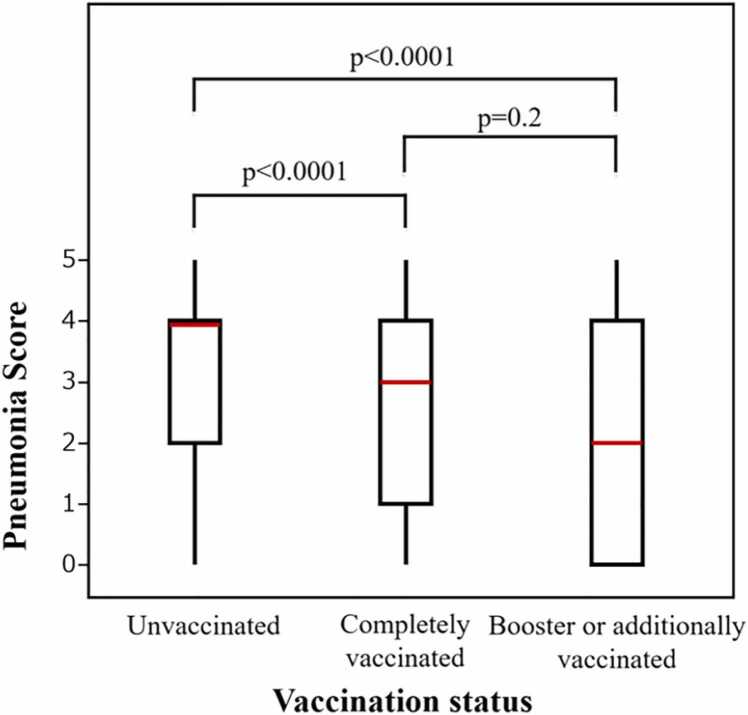
Comparison of Pneumonia Scores for each vaccination group.

**Fig. 4:**
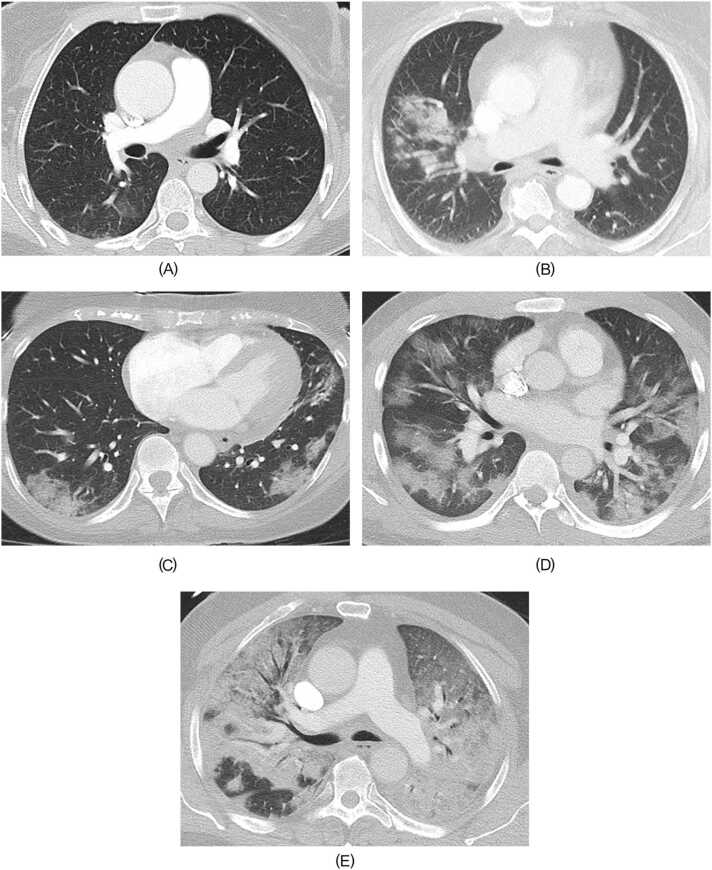
Representative cases for Pneumonia Score 1–5 A. Pneumonia Score 1. 39-year-old woman with hypertension, who had booster or additional vaccination. The time from last vaccination to COVID-19 diagnosis was 4.7 months. Initial CT was performed five days after diagnosis. Subtle GGOs were confirmed in right lower lobe. B. Pneumonia Score 2. 55-year-old woman with asthma, who had complete vaccination series. The time from last vaccination to COVID-19 diagnosis was 6 months. Initial CT scan was performed one day after diagnosis. Focal GGOs and parenchymal consolidation were confirmed in right upper lobe. C. Pneumonia Score 3. 62-year-old woman with a history of lung cancer treatment, who had complete vaccination series. The time from last vaccination to COVID-19 diagnosis was 4.7 months. CT performed eight days after diagnosis showed multifocal bilateral parenchymal consolidation and GGOs, which is consistent with non-extensive OP pattern. Follow- up CT about 2 months later showed that the consolidation and GGOs have mostly resolved. D. Pneumonia Score 4. 29-year-old man without any comorbidities, who had no vaccination. CT performed on the same day of COVID-19 diagnosis showed multifocal and extensive GGOs or consolidations accompanying focal spared areas, which is consistent with extensive OP pattern. E. Pneumonia Score 5**.** 69-year-old man with hypertension, who had no vaccination. CT performed on the same day of COVID-19 diagnosis showed diffuse and extensive consolidations and GGOs predominantly in dependent lung region accompanying focal spared areas in right lower lobe, which is consistent with DAD pattern. GGOs, ground-glass opacities; OP, organizing pneumonia; DAD, diffuse alveolar damage.

Interobserver agreement of the Pneumonia Score was high (weighted kappa score = 0.875). The unvaccinated group had a higher Pneumonia Score [median 4 (IQR 2–4)] than the completely vaccinated group [median 3 (IQR 1–4)] and booster or additionally vaccinated group [median 2 (IQR 0–4)], respectively (P < 0.0001 and P < 0.0001, respectively) (Fig. 3). There was no significant difference in Pneumonia Score between the completely vaccinated group and booster or additionally vaccinated group (P = 0.2). Among the unvaccinated, completely vaccinated, and booster or additionally vaccinated groups, the proportion of Pneumonia Score 0 was 13 % (16/124), 21 % (25/117), and 27 % (17/62), respectively, and the proportion of Pneumonia Score 5 was 14 % (17/124), 7 % (8/117), and 8 % (5/62), respectively (Table 3). Representative cases of Pneumonia Score 1–5 are shown in Fig. 4.

**Table 3:** Pneumonia Score and Vaccination status (n = 303).

Pneumonia score	Unvaccinated (n = 124)	Completely vaccinated (n = 117)	Booster or additionally vaccinated (n = 62)
**0**	16 (13 %)	25 (21 %)	17 (27 %)
**1**	4 (3 %)	12 (10 %)	11 (18 %)
**2**	12 (9 %)	18 (15 %)	11 (18 %)
**3**	22 (18 %)	27 (23 %)	5 (8 %)
**4**	53 (43 %)	27 (23 %)	13 (21 %)
**5**	17 (14 %)	8 (7 %)	5 (8 %)

### 3.5 Association of pneumonia score with vaccination status

A multivariable linear regression analysis of Pneumonia Score on vaccination status, adjusted for age, sex, BMI, smoking history, and comorbidities, was performed (Table 4 and S5). The completely vaccinated and booster or additionally vaccinated patients on average had a higher Pneumonia Score than the unvaccinated patients (P = 0.0005 and P < 0.0001, respectively), after adjusting for these common confounders (Table 4). However, there was no significant difference in Pneumonia Score between the booster or additionally vaccinated patients and completely vaccinated patients (P = 0.085) (Table S5). Male patients tended to have higher Pneumonia Score (P = 0.012) than female patients.

**Table 4:** Multiple linear regression analysis of Pneumonia Score on vaccination status, age, sex, BMI, smoking history, and comorbidities.

	Estimate	Standard error	t value	P value
Vaccination status Completely vaccinated Booster or additionally vaccinated (Ref: Unvaccinated)	-0.394 -0.622	0.111 0.140	-3.54 -4.43	0.0005 < 0.0001
Age	0.011	0.007	1.55	0.12
Sex Male (Ref: Female)	0.252	0.099	2.54	0.012
BMI	0.007	0.011	0.67	0.50
Smoking history Former/Current (Ref: Never/Unknown)	-0.084	0.100	-0.84	0.40
Lung disease	-0.060	0.106	-0.56	0.57
HT	0.090	0.108	0.84	0.40
CAD	-0.066	0.157	-0.42	0.67
DM	0.167	0.111	1.51	0.13
CKD	-0.042	0.156	-0.27	0.79
Malignancy	-0.061	0.112	-0.55	0.59

Ref; reference; BMI, body mass index; HT, hypertension; CAD, coronary artery disease; DM, diabetes mellitus; CKD, chronic kidney disease

## 4 Discussion

Previous studies have reported that a COVID-19 booster dose provided strong protection against infection, emergency department and urgent care encounters, hospitalization, severe illness, and death in adults during the period predominantly comprised of Delta and/or Omicron variants [3], [4], [5], [19], [20], [21], [22] and that CT severity played a role of an imaging surrogate of COVID-19 disease severity [23], [24], [25], [26]. Therefore, our study focused on the CT severity of unvaccinated patients and of vaccinated patients, including a booster or additional dose, during this period. For this, we gathered demographic and clinical characteristics and examined pneumonia severity on CT scans performed within a 6-week window around the time of diagnosis (−2 to +4 weeks), inclusive of inpatients and outpatients, who were diagnosed COVID-19. The cutoff of 2 weeks before diagnosis was determined with reference to a previous study that examined test results within 14 days after an initial negative result and reported the RT-PCR false-negative rate of 9.3 % [27]. The booster or additionally vaccinated and completely vaccinated groups had a lower Pneumonia Score than the unvaccinated group [median 2 (IQR 0–4), median 3 (IQR 1–4), and median 4 (IQR 2–4), respectively]. The differences were confirmed by robust results from the multivariable linear regression analysis. In addition, Pneumonia Score in the booster or additionally vaccinated group tended to be lower than in the completely vaccinated group, though not statistically significance. Analysis of clinical outcomes also showed that the booster or additionally vaccinated patients had significantly shorter hospital stays and a lower probability of hospitalization and ICU admission than the unvaccinated patients.

Recent studies have examined the difference of CT findings in the acute phase of COVID-19 between unvaccinated patients and vaccinated patients [14], [15], [16]. Lee et al. reported that the proportion of patients with negative CT scan was significantly greater in the fully vaccinated group than in the unvaccinated group [15]. Verma et al. reported that the mean CT severity score was significantly lower in the completely vaccinated patients in comparison to the incompletely vaccinated and non-vaccinated patients [16]. Despite differences such as study time window, predominant COVID-19 variants, scoring system of CT severity, and type of vaccine, the finding that CT pneumonia severity of vaccinated patients is lower than unvaccinated patients appear to be consistent. One example is Tsakok et al. who further compared CT severity in booster vaccinated patients with single/double vaccinated or unvaccinated patients and reported that patients who had received a booster dose had lower chest CT severity score than unvaccinated patients. In addition, there was a significantly greater proportion of patients who were categorized as normal in the booster or single/double vaccinated patients compared to unvaccinated patients [14]. The results of our study were consistent with the previous studies, and support vaccine effectiveness against the pulmonary complications of COVID-19.

We also found that male sex was an independent risk factor for a higher Pneumonia Score, representing CT severity; while males and females were both at risk of COVID-19 infection, male sex was associated with more severe disease outcomes, such as ICU admission and death [28], [29], [30], possibly due to biological differences between sexes in the immune response to infection [28].

An additional primary dose for immunocompromised persons and a booster dose for those aged 65 years and older, were initiated because of reduced immunogenicity in immunocompromised persons, waning vaccine effectiveness over time, and the highly transmissible Delta variant [17]. In the present study, a greater proportion of the booster or additional vaccinated group had older age and underlying comorbidities and might reflect the vaccination policy. As of July 26, a second booster dose for adults ages 50 years and older and some people ages 12 years and older who are moderately or severely immunocompromised are recommended [31], and further studies including these subjects would be valuable.

There are several limitations to this study. First, there might be selection biases. Hundreds of COVID-19 patients were excluded because of a lack of chest CT data within the -2 to 4 weeks window around diagnosis. Patients with no scans were slightly younger, more likely to be vaccinated and also more likely to be cancerous than those with scans. Second, the timing of CT evaluation was not strictly consistent as we used a 6-week window around the time of diagnosis (−2 to +4 weeks). A previous study reported that four stages of evolution on chest CT were identified from symptom onset: early stage (0–4 days), progressive stage (5–8 days), peak stage (9–13 days), and absorption stage (≥14 days) in COVID-19 patients [9]. Although evolving changes in the lungs may affect the evaluation of CT severity, the median interval between diagnosis and CT with Pneumonia Score was short at 2 (IQR 0–10) days in this study, which made it possible to evaluate CT findings in the acute phase. Third, our study may include patients who had incidentally tested RT-PCR positive of SARS-CoV-2 and had other respiratory illnesses. Finally, there may be an issue of generalizability. Our data came from a single academically based institution, which may not well represent a general population. For example, the institution may present more vaccinated or Caucasian patients than the general population. Caution must be exercised when interpreting and generalizing the results.

In conclusion, COVID-19 pneumonia severity on CT scans, as an imaging surrogate for disease severity, showed significant differences between vaccinated and unvaccinated patients. Among vaccinated patients, including those with booster or additional doses, CT severity was milder than that among unvaccinated patients during the Delta and Omicron variant predominant period. This study supports vaccine effectiveness against COVID-19 from a radiological perspective.

## Funding

M.N. is supported by 10.13039/100000002NIH (R01CA203636, U01CA209414, R01HL111024, and R01CA240592); Y.L. is supported by 10.13039/100000054NCI (R01CA249096); B.M. is supported by NIH (R01EB030470); B.D.L. is supported by NIH/NHLBI (1OT2HL162087); K.M.K. is supported by NIH (R01AI150575, R01AI165382, and R01DE025208); H.H. is supported by NIH (R01CA203636, 5U01CA209414, and R01HL135142), NIH/NHLBI (R01HL111024 and R01HL130974); D.C. is supported by NIH (5U01CA209414).

## CRediT authorship contribution statement

**Noriaki Wada:** Conceptualization, Methodology, Formal analysis, Investigation, Data curation, Writing - Original draft. **Yi Li:** Methodology, Formal analysis, Writing - Review & Editing. **Takuya Hino:** Conceptualization, Methodology, Formal analysis, Investigation, Data curation, Writing - Review & Editing. **Staci Gagne:** Methodology, Writing - Review & Editing. **Vladimir I. Valtchinov:** Resource, Data curation, Writing - Review & Editing. **Elizabeth Gay:** Writing - Review & Editing. **Mizuki Nishino:** Methodology, Investigation, Writing - Review & Editing. **Bruno Madore:** Methodology, Writing - Review & Editing. **Charles R. G. Guttmann:** Writing - Review & Editing. **Sheila Bond:** Methodology, Writing - Review & Editing. **Kousei Ishigami:** Writing - Review & Editing. **Gary M. Hunninghake:** Methodology, Writing - Review & Editing. **Bruce D. Levy:** Writing - Review & Editing. **Kenneth M. Kaye:** Methodology, Writing - Review & Editing. **David C. Christiani:** Methodology, Resource, Writing - Review & Editing. **Hiroto Hatabu:** Conceptualization, Methodology, Formal analysis, Investigation, Resources, Project administration, Supervision, Writing - Review & Editing.

## Ethical statement

This study was approved by the institutional review board of Brigham and Women’s Hospital. Written informed consents were obtained from all the participants.

## Conflict of interest

M.N. reports research grant to the institution from Merck, Canon Medical Systems, AstraZeneca, and Daiichi Sankyo; and consulting fees from Daiichi Sankyo and AstraZeneca, outside the submitted work. C.R.G.G. reports stock ownership of GSK, Roche, and Novartis, outside the submitted work. G.M.H. reports consulting fees from Boehringer Ingelheim, Gerson Lehrman Group, and Chugai Pharmaceuticals, outside the submitted work. B.D.L reports grants or contacts from NIH, Pieris Pharmaceuticals, SRA, and Sanofi; royalties or licenses from Propeller Health; consulting fees from AstraZeneca, NControl, Cartesian, Novartis, Gossamer Bio, and Thetis Pharmaceuticals; participation on a data safety monitoring board or advisory board from NIAID; and stock or stock options from Entrinsic Biosciences and Nocion Therapeutics, outside the submitted work. H.H. reports grants or contracts from Canon Medical Systems Inc and Konica-Minolta Inc; and consulting fees from Canon Medical Systems Inc and Mitsubishi Chemical Co, outside the submitted work. The other authors have no conflicts of interest to be disclosed related to this article.
