# Supplementary material for: COVID-19 Vaccination reduced pneumonia severity
Source: Eur J Radiol Open. 2022 Nov 11;9:100456. doi: 10.1016/j.ejro.2022.100456 (PMC9650570; doi:10.1016/j.ejro.2022.100456)
Supplement: Supplementary file 1 — Supplementary material. [file mmc1.docx]

**Supplementary material**

**Supplementary Results**

The vaccination status and the demographic and comorbidity conditions between those with and without chest scans were compared (Table S1). In general, these two populations were comparable, except that patients with no scans were slightly younger and more likely to be vaccinated. Interestingly, patients with no scans were also more likely to be cancerous (44% vs 31%) than those with scans.

**Table S1. Comparison of demographic/clinical data of patients with and without CT scans**

|  | **Patients with CT scans**  **(n=303)** | **Patients without CT scans**  **(n=439)** | **P value** |
| --- | --- | --- | --- |
| Vaccination status  Unvaccinated  Completely vaccinated  Booster or additionally vaccinated | 124 (41%)  117 (39%)  62 (20%) | 60/415 (14%)^a^  210/415 (51%)^a^  145/415 (35%)^a^ | <0.0001 |
| Age | 59.4±16.3 | 56.1±17.5 | 0.011 |
| Sex  Male  Female | 125 (41%)  178 (59%) | 172 (39%)  267 (61%) | 0.59 |
| BMI | 30.8±9.4 | 30.5±9.0 | 0.64 |
| Smoking history  Former/Current  Never/Unknown | 148 (49%)  155 (51%) | 212 (48%)  227 (52%) | 0.94 |
| Lung disease | 114 (38%) | 161 (37%) | 0.82 |
| HT | 170 (56%) | 253 (58%) | 0.71 |
| CAD | 37 (12%) | 73 (17%) | 0.11 |
| DM | 89 (29%) | 96 (22%) | 0.025 |
| CKD | 36 (12%) | 65 (15%) | 0.28 |
| Malignancy | 95 (31%) | 194 (44%) | 0.0004 |

^a^ Vaccination data was unknown (n=24).

**Table S2. Demographic data and clinical information of the whole cohort (n=303)**

| **Parameter** | **Value** |
| --- | --- |
| Age (years) | 59.4±16.3 |
| Sex (Male:Female) | 125:178 |
| BMI (kg/m^2^) | 30.8±9.4 |
| Smoking history  Never  Former/Current  Unknown | 144/303 (48%)  148/303 (49%)  11/303 (3%) |
| Vaccination status  Unvaccinated  Completely vaccinated  Booster or additional vaccinated | 62/303 (20%)  117/303 (39%)  124/303 (41%) |
| Laboratory findings |  |
| CRP (mg/L) (n=210) | 97.2 (35-174) |
| D-dimer (ng/mL) (n=226) | 1465 (820-3612) |
| Comorbidities |  |
| Any comorbidities | 255/303 (84%) |
| Lung disease | 114/303 (38%) |
| HT | 170/303 (56%) |
| CAD | 37/303 (12%) |
| DM | 89/303 (29%) |
| CKD | 36/303 (12%) |
| Malignancy | 95/303 (31%) |
| Clinical outcome |  |
| Hospitalization | 235/303 (78%) |
| Hospital stays (days) | 5 (1-13) |
| ICU admission | 51/303 (17%) |
| All-cause death | 54/303 (18%) |
| Interval from diagnosis to CT (days) | 2 (0-10) |

BMI, body mass index; CRP, C-reactive protein; HT, hypertension; CAD, coronary artery disease;

DM, diabetes mellitus; CKD, chronic kidney disease; ICU, intensive care unit

**Table S3. Vaccine type in completely vaccinated group (n=117)**

|  | **Completely vaccinated**  **(n=117)** |
| --- | --- |
| Two doses  BNT162b2  mRNA-1273 | 61 (52%)  38 (32%) |
| One dose |  |
| Ad26.COV2.S | 18 (15%) |

**Table S4. Vaccine type in booster or additionally vaccinated group (n=62)**

|  | **Booster or additionally vaccinated**  **(n=62)** |
| --- | --- |
| Three doses  BNT162b2 | 38 (61%) |
| mRNA-1273 | 18 (29%) |
| mRNA-1273 and BNT162b2 | 2 (3%) |
| Two doses  Ad26.COV2.S | 2 (3%) |
| Ad26.COV2.S and BNT162b2 or mRNA-1273 | 2 (3%) |

**Table S5. Multiple linear regression analysis of Pneumonia Score on vaccination status, age, sex, BMI, smoking history, and comorbidities**

|  | **Estimate** | **Standard error** | **t value** | **P value** |
| --- | --- | --- | --- | --- |
| Vaccination status  Booster or additionally vaccinated  Unvaccinated  (Ref: Completely  vaccinated) | -0.228  -0.394 | 0.132  0.111 | -1.73  -3.54 | 0.085  0.0005 |
| Age | 0.011 | 0.007 | 1.55 | 0.12 |
| Sex  Male  (Ref: Female) | 0.252 | 0.099 | 2.54 | 0.012 |
| BMI | 0.007 | 0.011 | 0.67 | 0.50 |
| Smoking history  Former/Current  (Ref: Never/Unknown) | -0.084 | 0.100 | -0.84 | 0.40 |
| Lung disease | -0.060 | 0.106 | -0.56 | 0.57 |
| HT | 0.090 | 0.108 | 0.84 | 0.40 |
| CAD | -0.066 | 0.157 | -0.42 | 0.67 |
| DM | 0.167 | 0.111 | 1.51 | 0.13 |
| CKD | -0.042 | 0.156 | -0.27 | 0.79 |
| Malignancy | -0.061 | 0.112 | -0.55 | 0.59 |

Ref; reference; BMI, body mass index; HT, hypertension; CAD, coronary artery disease;

DM, diabetes mellitus; CKD, chronic kidney disease
